# Cool Excimer Laser-Assisted Angioplasty vs. Percutaneous Transluminal Angioplasty for Infrapopliteal Arterial Occlusion: A Meta-Analysis and Systematic Review

**DOI:** 10.3389/fcvm.2021.783358

**Published:** 2022-02-02

**Authors:** Mi Zhou, Lixing Qi, Yongquan Gu

**Affiliations:** Department of Vascular Surgery, Xuanwu Hospital, Capital Medical University, Beijing, China

**Keywords:** cool excimer laser-assisted angioplasty, percutaneous transluminal angioplasty, infrapopliteal arterial occlusion, peripheral arterial disease, endovascular intervention

## Abstract

**Background:**

Percutaneous transluminal angioplasty (PTA) has been the conventional therapy to infrapopliteal arterial occlusion. Lately, cool excimer laser-assisted angioplasty has been proposed to be the alternate methods. We performed a systematic review and meta-analysis of prospective and retrospective cohort studies and randomized controlled trials to assess the effect of cool excimer laser-assisted angioplasty vs. tibial balloon angioplasty in patients with infrapopliteal arterial occlusion.

**Methods and Results:**

We systematically searched PubMed, Embase, Cochrane Central Register of Controlled Trials (CENTRAL) (all up to April, 2021). All prospective and retrospective cohort studies and randomized controlled trials comparing clinical outcomes between cool excimer laser-assisted angioplasty and tibial balloon angioplasty were included. The main endpoints were amputation-free survival (AFS), primary patency (6 months and 12 months) and free from target lesion revascularization (TLR) (3 years). Secondary outcomes included the major amputation (1 year), dissection, embolization and bailout stent. We chose the effect model according to studies' heterogeneity. A total of 122 articles were found. According to inclusion criteria, 6 papers were finally selected for the detailed evaluation. Of the 6 papers, 4 were prospective cohort studies, and 2 were retrospective studies. Compared with PTA, CELA significantly increased the rate of patency (6 months: MD 13.01, 95% CI 3.12-22.90, *P* < 0.05; 12 months: MD 11.88 95% CI 8.38-15.37, *P* < 0.05) and the rate freedom from TLR (36 months: MD 7.51 95% CI 0.63-14.40, *P* < 0.05). There is no statistically difference of AFS, major amputation, dissection, embolization and bailout stent between CELA group and PTA group (MD −2.82, 95% CI −8.86-3.22, *P* = 0.36; MD −0.17, 95% CI −1.04-0.70, *P* = 0.39; MD 1.11, 95% CI 0.58-2.10, *P* = 0.75; MD 0.46, 95% CI 0.11-1.99, *P* = 0.30; MD 1.89, 95% CI 0.92-3.88, *P* = 0.09).

**Conclusions:**

CELA had superior clinical (freedom from TLR) and angiographic outcomes (patency rate) for infrapopliteal arterial occlusion at the same time CELA does not have increased intervention-related complications compared to PTA. However, CELA is unable to improve the patient's limb salvage rate compared with PTA.

## Introduction

Infrapopliteal peripheral arterial disease (PAD) has a high rate of limb loss within 6 months when untreated (1, 2). The overall morbidity of PAD is in the range of 3-10%, and increases to 15-20% in persons over 70 years. With the trend of the aging, the social and economic burden of PAD is considerable. Endovascular intervention, such as angioplasty, debulking, and stenting offer a less invasive method, but evidence of safety and efficacy is heterogenous (3). Infrapopliteal arterial disease is often accompanied by extensive severe calcification, lumen narrowing, and poor distal outflow tracts, significantly increasing the difficulty of endovascular therapy and resulting in high likelihood of restenosis and the need of revascularization.

Recent reports have indicated considerable results of angioplasty for the treatment of infrapopliteal artery occlusion and benefited equivalent clinical limb salvage rates to bypass surgery (4). However, plain of balloon angioplasty (POBA) has a high rate of reocclusion, revascularization and complications (embolization, dissection, bailout stenting). Theoretically, excimer laser removes biologic tissue by photochemical desorption and the injected energy would convert the tissue to vaporized fragments (5). Cool excimer laser-assisted angioplasty (CELA) can decrease the rate of residual stenosis, thromboembolization, perforation to some extent (6). Traditionally, underdeveloped application of laser resulted in poor immediate and mid-term outcomes (7, 8), while recent studies have reported satisfactory clinical outcomes in high-volume centers with refinement of laser techniques (1, 9). However, clinical efficacy of CELA for infrapopliteal arterial occlusion is inconclusive.

We aim to determine the safety and efficacy of CELA for infrapopliteal arterial occlusion by carried out this meta-analysis of prospective and retrospective cohort studies and randomized controlled trials.

## Materials and Methods

Our study was conducted according to the Preferred Reporting Items for Systematic Reviews and Meta-Analysis (PRISMA) guidelines for meta-analysis (Preferred Reporting Items for Systematic Reviews and Meta-Analyses: The PRISMA Statement).

### Search Strategy and Inclusion Criteria

All relevant studies published until April 2021 were searched in PubMed, Embase, and the Cochrane Central Register of Controlled Trials (CENTRAL) without language restrictions. The following subject headings and keywords: “Laser” OR “laser angioplasty” OR “laser angioplasties” OR “angioplasties” OR “repair, endoluminal” OR “infrapopliteal” and “popliteal” OR “infragenicular” OR “percutaneous transluminal angioplasty” OR “transluminal angioplasty” and “percutaneous transluminal angioplasty” OR “endoluminal repair” et al. We contacted the corresponding author of the article to obtain related information not available from the dataset.

### Inclusion and Exclusion Criteria

Inclusion criteria were (1) prospective and retrospective cohort studies and randomized controlled trials; (2) patients with infrapopliteal occlusion; (3) treatment methods were CELA or PTA; (4) a minimum follow-up of 6 months.

### Endpoints and Data Extraction

The primary study endpoints refer to the endpoints that are directly related to the main purpose of the clinical therapy and provide the most clinically meaningful and convincing evidence. The secondary study endpoints are supportive of the primary endpoints. The secondary endpoints can be considered as an auxiliary support for the benefit of patients when the primary endpoints cannot be completed due to a long observation time.

The primary study endpoints were primary patency (6 months and 12 months), free from TLR (3 years) and amputation-free survival (AFS). The secondary endpoints included the major amputation (1 year), bailout stenting, dissection, embolization.

**Figure 1:**
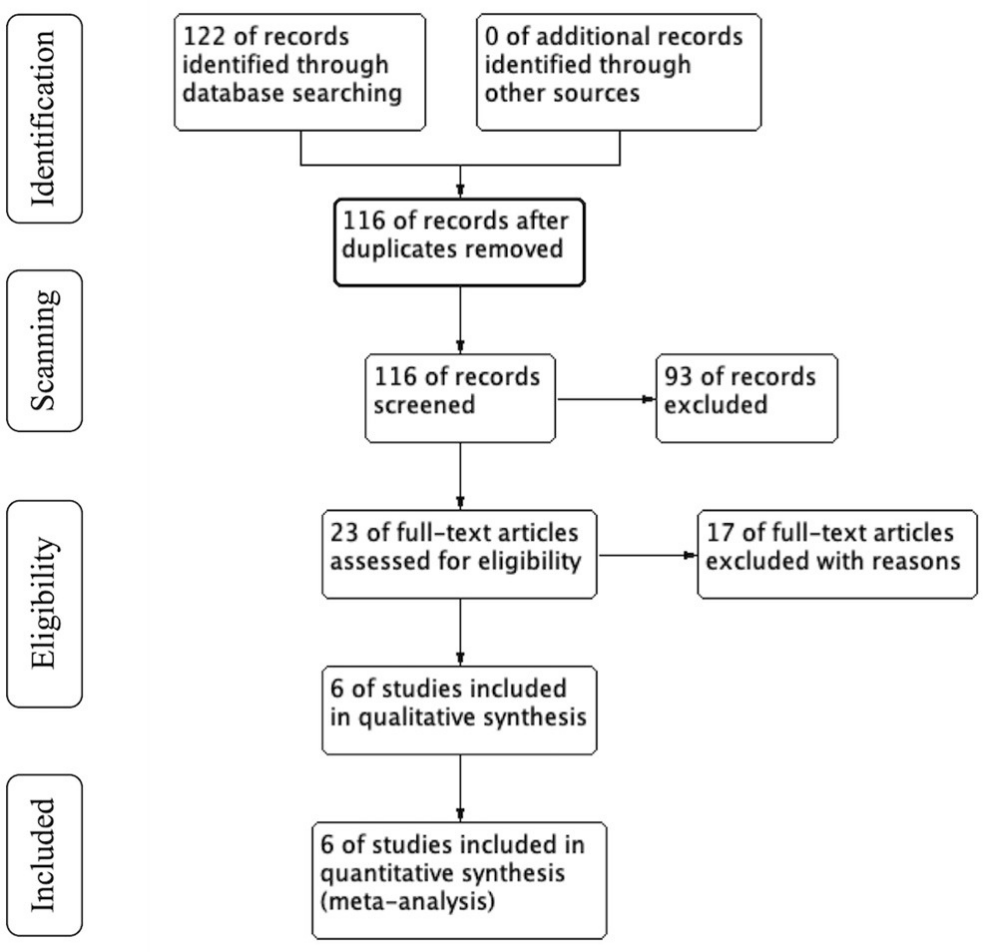
PRISMA flow chart.

Data were extracted from each selected study: name of the first author, study design, patient characteristics (basic disease, age, number of patients and sex ratio, ABI, TASC class). The details are shown in Figure 1. Data search and extraction, risk of bias was performed by two reviewers independently in accordance with the PRISMA recommendations. We assessed the risk of bias of prospective and retrospective studies according to Effective Practice and Organization of Care (EPOC) criteria.

### Data Synthesis and Statistical Analysis

Mean difference (MD) with 95% confidence intervals (CIs) and Odds ratio (OR) with 95% CIs were used to summarize the continuous and dichotomous variables, respectively. We used the Cochran's Q-statistic test and the I^2^ test to assess the heterogeneity between studies, with I^2^ more than 50% and *p* < 0.05 suggesting significantly heterogeneity, a random-effects model was adopted. Otherwise, we adopted a fixed effects model to evaluate the overall effect. Subgroup analysis were performed based on the following factors: publication year, region and samples size. Sensitivity analysis was performed through studies exclusion one by one. Review Manager Version (version 5.4; Cochrane Collaboration, Copenhagen, Denmark) was used to perform statistical analysis.

## Results

### Study Selection

Initially, 122 articles were obtained by searching with the proposed input, and 116 articles were retrieved after removing duplicates. 93 articles were initially excluded after reading the titles and abstracts because they did not meet the inclusion criteria or met the exclusion criteria. A total of 23 articles was investigated, and 17 of them were excluded by reading full-text. Finally, 6 articles were included in the meta-analysis (3, 6, 10–13). Figure 1 shows the process of literature search in detail.

### Study Characteristics

A total 6 studies with 2,217 participants were included in meta-analysis. Most of the included patients suffer from hypertension, diabetes, hyperlipidemia and coronary artery disease. More details of patients' clinical characteristics are showed in Table 1.

**Table 1:** Characteristics of included trials.

**References**	**Design**		**Hypertension**	**DM**	**CAD**	**Dyslipidemia**	**Age, y**	***n***	**Male gender**	**ABI**	**TASC class**
Sultan et al. (6) (PMID:23448976)	Single center, prospective, controlled	CELA	26 (62%)	32 (77%)	15 (37%)	32 (77%)	68 (52~86)	42	22 (52%)	0.35	B = 5 (11.9%); C = 13 (30.9%); D = 18 (42.8%)
		PTA	32 (69%)	38 (82%)	16 (34%)	36 (77%)	70 (48~96)	47	25 (53%)	0.39	B = 5 (10.6%); C = 13 (27.6%); D = 18 (38.3%)
Steinkamp et al. (10) (PMID:12546591)	Single center, prospective, nonrandomized, controlled	CELA	52 (40.9%)	35 (27.5%)		23 (18.1%)	64.0 ± 9.7 (49~86)	127	70 (55%)	0.34 ± 0.16	A/B = 99 (78%); C = 17 (13.4%); D = 11 (8.7%)
		PTA	42 (47.7%)	21 (23.8%)		18 (20.4%)	62.0 ± 8.7 (48~83)	88	52 (59%)	0.33 ± 0.18	A/B = 74 (84.1%); C = 10 (11.4%); D = 4 (4.5%)
Bosiers et al. (11) (PMID:16956473)	Dual-center, prospective, nonrandomized, controlled	CELA						64			
		PTA						79			
Piyaskulkaew et al. (3) (PMID:26489379)	Single center, prospective, nonrandomized, controlled	CELA	372 (96.1%)	230 (59.6%)	273 (70.5%)	368 (95.1%)	71.59 ± 11.117	395	211 (53.4%)		A/B = 4 (1%); C = 26 (6.6%); D = 365 (92.4%)
		PTA	320 (97.6%)	188 (57.3%)	223 (68%)	314 (95.7%)	71.14 ± 11.705	331	182 (55%)		A/B = 0; C = 111 (33.5%); D = 220 (66.5%)
Kokkinidis et al. (12) (PMID:32952073)	Dual-center, retrospective, nonrandomized, controlled	CELA	66 (87%)	49 (64%)	33% (25/76)	153.8 (51.1%)	69.5± 9.86	76	57 (75%)	0.36	TASC C/D = 47 (82%)
		PTA	204 (86%)	186 (78%)	52% (122/234)	135.9 (44.3%)	70.1± 12.7	237	160 (67.5%)	0.25	TASC C/D = 53 (45%)
Singh et al. (13) (PMID:24155171)	Single-center, retrospective, nonrandomized, controlled	CELA	374 (96.1%)	232 (59.8%)	70.7% (275/398)	370 (95.1%)	71.6 ± 11.1	398	213 (53.5%)		A/B = 4 (1%); C = 26 (6.5%); D = 368 (92.5%)
		PTA	322 (97.6%)	190 (57.6%)	67.9% (224/333)	316 (95.8%)	71.1 ± 11.8	333	183 (55 %)		A/B = 0; C = 111 (33.3%); D = 222 (66.7%)

*DM, Diabetes mellitus; CAD, Coronary heart disease; ABI, Ankle brachial index; TASC class, Trans-Atlantic Inter-Society Consensus committee class*.

### Risk of Bias and Quality Assessment

**Figure 2:**
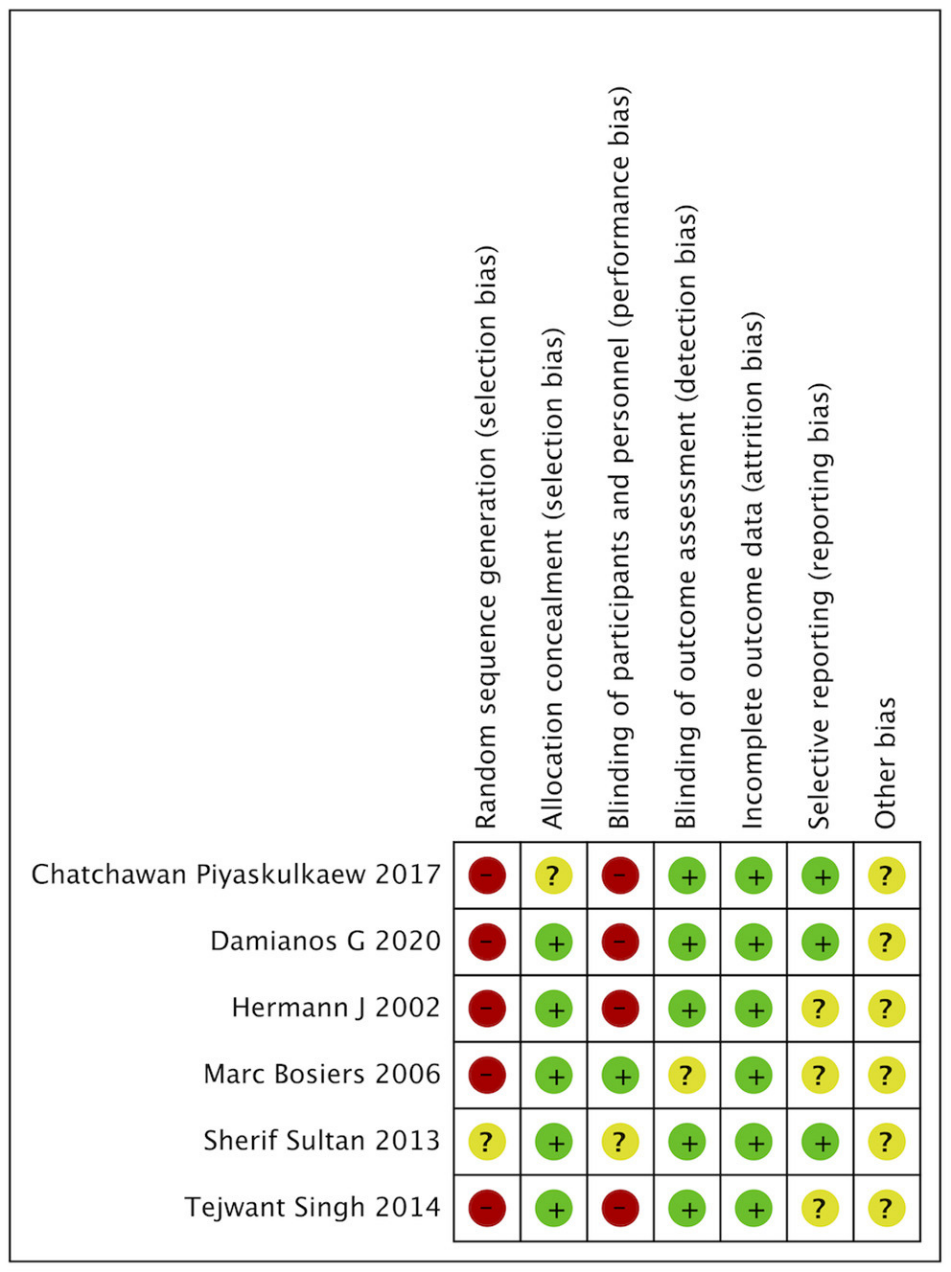
Risk of bias summary. “+” refers to low risk of bias, “–” refers to high risk of bias, “?” refers to unclear risk of bias.

The risk-of-bias assessment of included prospective and retrospective studies was assessed according to the Cochrane Collaboration's tool and summarized in Figures 1, 2, respectively. The risk of bias on study outcomes is typically judged by the researchers according to an assessment of the quality of each individual study from the aspects of sampling bias, selection bias, and intra-study bias.

### Primary Study Endpoints

#### Patency

**Figure 3:**
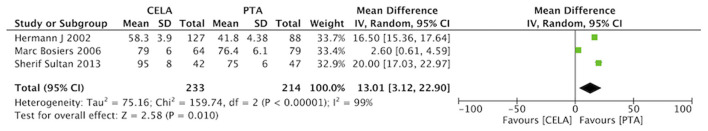
Forest plot for 6-month patency. Random, a randomized effects model; CI, confidence intervals. The green squares stand for the weight of studies in meta-analysis. Black rhomboid represents the effects of combination of above three studies.

Three studies reported the artery patency of target lesion at 6 months follow-up. The patency of CELA group was higher than the PTA group (MD 13.01, 95% CI 3.12-22.90, *P* = 0.01) using a random effects model (I^2^ = 99%, *P* < 0.00001) (Figure 3).

**Figure 4:**
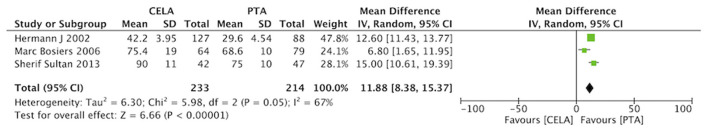
Forest plot for 1-year patency. Random, a randomized effects model; CI, confidence intervals. The green squares stand for the weight of studies in meta-analysis. Black rhomboid represents the effects of combination of above three studies.

Three studies reported the artery patency of target lesion at 1 year follow-up. The patency of CELA group was higher than the PTA group (MD 11.88, 95% CI 8.38-15.37, *P* < 0.00001) using a random effects model (I^2^ = 67%, *P* = 0.05) (Figure 4).

#### Freedom From TLR

**Figure 5:**
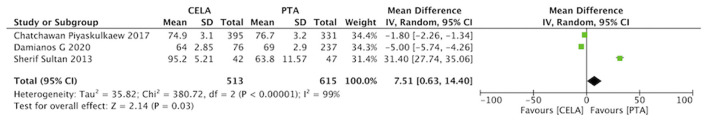
Forest plot for freedom from TLR at 3 years. Random, a randomized effects model; CI, confidence intervals; TLR, target lesion revascularization. The green squares stand for the weight of studies in meta-analysis. Black rhomboid represents the effects of combination of above three studies.

Three studies reported freedom from TLR at 3 years follow-up. The rate of freedom from TLR in the CELA group was significantly higher than the PTA group (MD 7.51, 95% CI 0.63-14.40, *P* < 0.00001) using a random effects model (I^2^ = 99%, *P* = 0.03) (Figure 5).

#### Amputation-Free Survival

**Figure 6:**
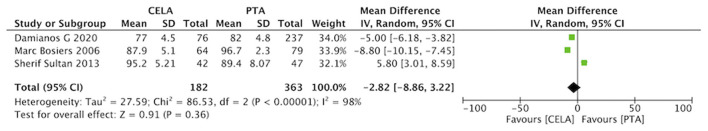
Forest plot for amputation-free survival (AFS). Random, a randomized effects model; CI, confidence intervals. The green squares stand for the weight of studies in meta-analysis. Black rhomboid represents the effects of combination of above three studies.

Three studies reported amputation-free survival at 1 year follow-up. The rate of amputation-free survival was no statistical difference between CELA group and the PTA group (MD −2.82, 95% CI −8.86-3.22, *P* < 0.00001) using a random effects model (I^2^ = 98%, *P* = 0.36) (Figure 6).

### The Secondary Study Endpoints

#### Major Amputation

**Figure 7:**
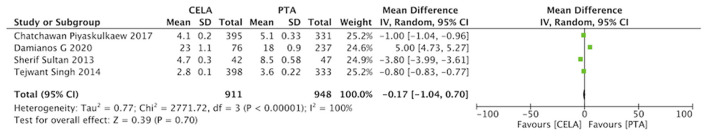
Forest plot for major amputation at 1 year. Random, a randomized effects model; CI, confidence intervals. The green squares stand for the weight of studies in meta-analysis. Black rhomboid represents the effects of combination of above four studies.

Four studies reported major amputation at 1 year follow-up. The rate of major amputation was no statistical difference between CELA group and the PTA group (MD −0.17, 95% CI −1.04-0.70, *P* < 0.00001) using a random effects model (I^2^ = 100%, *P* = 0.70) (Figure 7).

#### Bailout Stenting

**Figure 8:**
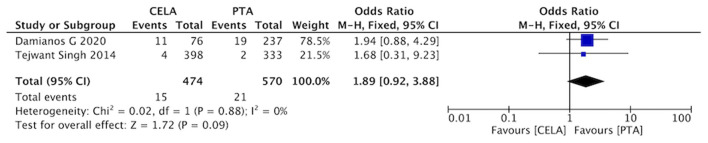
Forest plot for bailout stenting. Fixed, a fixed effects model; CI, confidence intervals. The blue squares stand for the weight of studies in meta-analysis. Black rhomboid represents the effects of combination of above two studies.

Two studies reported bailout stenting during the process of surgery. The rate of bailout stenting was no statistical difference between CELA group and the PTA group (OR 1.89, 95% CI 0.92-3.88, *P* = 0.88) using a fixed effects model (I^2^ = 0%, *P* = 0.09) (Figure 8).

#### Dissection

**Figure 9:**
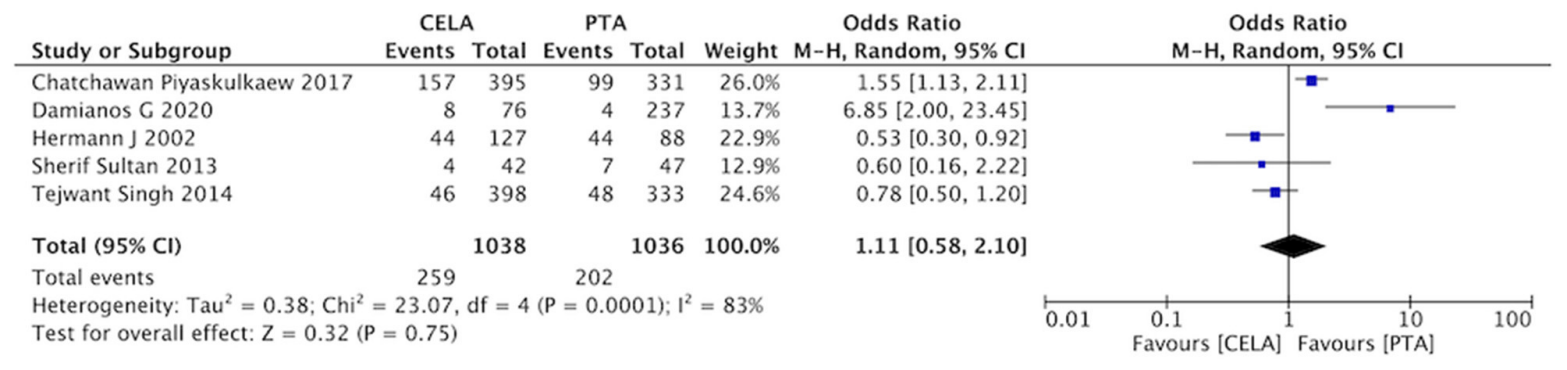
Forest plot for dissection. Random, a randomized effects model; CI, confidence intervals. The blue squares stand for the weight of studies in meta-analysis. Black rhomboid represents the effects of combination of above five studies.

Five studies reported vessel dissection during the process of surgery. The rate of dissection was no statistical difference between CELA group and the PTA group (OR 1.11, 95% CI 0.58-2.10, *P* = 0.0001) using a random effects model (I^2^ = 83%, *P* = 0.75) (Figure 9).

#### Embolization

**Figure 10:**
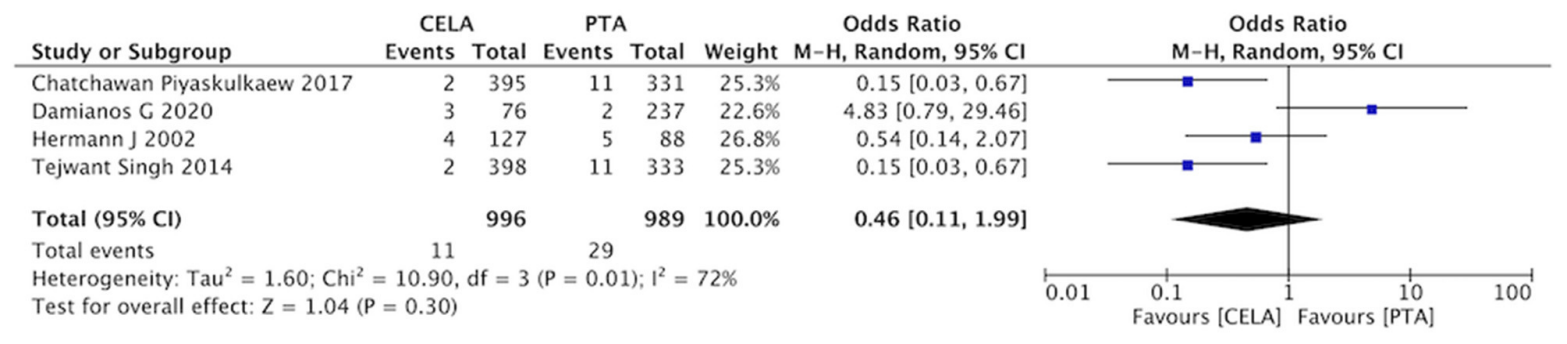
Forest plot for embolization. Random, a randomized effects model; CI, confidence intervals. The blue squares stand for the weight of studies in meta-analysis. Black rhomboid represents the effects of combination of above four studies.

Four studies reported ectopic embolization during the process of surgery. The rate of vessel embolization was no statistical difference between CELA group and the PTA group (OR 0.46, 95% CI 0.11-1.99, *P* = 0.01) using a random effects model (I^2^ = 72%, *P* = 0.30) (Figure 10).

## Discussion

Our meta-analysis evaluated the efficiency and safety of CELA therapy vs. PTA alone for treatment of patients suffering infrapopliteal artery occlusion disease for the first time. Comparing with conventional PTA alone, CELA therapy showed a significantly higher patency of 6 months and 1 year in target lesion, offered the advantages of an increase rate free from TLR, meanwhile not increased surgery complication (embolization, bailout stenting, dissection). However, CELA could not reduce major amputation and amputation-free survival. These data provide evidence that CELA maybe a more effective and safer therapetic strategy for infrapopliteal artery occlusion.

Laser-assist angioplasty was firstly applied for vascular intervention since the early 1980s in coronary disease (14). Initially, underdevelopment use of the laser resulted in high rate of complications (perforation, dissection, and thermal injure) (15, 16). With the advance of equipment and technology, recent studies have reported some convincing result supporting laser for the treatment of peripheral artery disease. There have many meta-analyses compare the efficacy and safety between laser-assist angioplasty and POBA in iliofemoral artery occlusion, however, no meta-analysis summarized laser effectiveness on infrapopliteal artery occlusion. Our finding verified showed that CELA therapy shows superior clinical outcome over PBOA.

The importance of infrapopliteal artery patency upon clinical outcomes was recently reported by some studies (17–20). Tibial arterial patency was mandatory during the process of wound healing (4, 21, 22). Baumann F observed a prolong time of wound healing in tibial restenosis patients and a significant corroboration with TLR and POBA (20). Target lesion revascularization was necessary in 48% of patients with restenosis of the tibial artery in 73% at 12-month follow-up. Restenosis was observed in 68.8 and 74% of limbs in CLI patients undergoing tibial artery POBA after 3 months and 1 year, respectively (23, 24). In our analysis, three studies recorded the patency of target vessel during 6-month and 12-month follow-up and 3-year freedom from TLR verified that CELA has a higher patency rate and free from TLR over POBA alone. This may be due to the excimer laser can selectively ablate atherosclerotic plaques and thrombi, while reducing subintimal angioplasty (25, 26). Our findings are in accordance with the results of the study of Laird et al. that CELA can be used for complex anatomic lesions with a high technical success and admirable short-term success rates (27).

However, our finding also verified that there is no advantage of AFS and major amputation rate to CELA compared with POBA alone. Higher patency of infrapopliteal artery did not translate into high limb salvage rate. This is very similar to other studies' conclusion. Michael C represented this result as a case-selection bias, accounting that CELA was not usually used on cases where a guidewire could not be successfully access to the occlusion lesion and suggested a randomized trial to verify the result (28). Our findings are in accordance with the results of the study of Romiti et al. that significant differences in patency rate did not result in significant difference in clinical outcomes (limb salvage) (4). Based on these findings, we assumed that the increased perfusion to the target lesion was mandatory to wound healing but not for keeping tissue integrity.

Theoretically, the excimer laser can vaporize intravascular plaques, effectively remove hyperplastic tissue (29), thereby reducing the risk of ectopic embolism. Laser atherectomy can reduce the probability of dissection after PTA, the implantation of bail-out stents and avoidance of subintimal technique. Previous studies reported embolism after PTA occurred in 2.6% of the cases (30) and laser atherectomy related distal embolization occurred in 3-10% of the cases (27, 31–35). However, our findings suggested that there is no significant advantage to CELA compare to POBA in surgery complications (dissection, embolization, bail-out stenting). We represented the result as case-selection bias. Patients with CELA therapy have more probabilities to accompany by severe calcifications. Calcification lesions are related to the dissection post-PTA, ectopic embolization and bailout stents, resulting in the finding that CELA has no obvious advantage in surgical complications compared with POBA. Without a randomized clinical trial, this result is difficult to address, but admittedly the datasets are not positive.

Limitations of our analysis included the retrospective studies other than Randomized Controlled Trial (RCT) trial and relatively small samples size. Retrospective nature could lead to various biases, resulting more determinants for outcome, however, currently there is no RCT study on laser treatment of the infrapopliteal artery. The relatively small samples size magnifies the result of analysis process. The current evidence is not sufficient to represent a real-world application of CELA technology for infrapopliteal artery occlusion revascularization, however, our findings tend to mirror contemporary revascularization studies.

## Conclusion

Our meta-analysis showed that CELA has superior patency rate (6 months and 12 months) and freedom from TLR. However, comparing to POBA therapy, CELA did not decrease amputation rate and AFS, meanwhile had no advantage in surgery complications. Considering the retrospective nature of our analysis and inherent bias shortcomings, there is no any indication for revascularization. Large-scale RCTs are needed to verify the safety and efficiency of CELA therapy to infrapopliteal artery occlusion.

## Data Availability Statement

The original contributions presented in the study are included in the article/supplementary material, further inquiries can be directed to the corresponding author/s.

## Author Contributions

MZ and YG designed the methods, analyzed the data, and results. MZ and LQ wrote the manuscript and prepared figures. All authors have read and agreed to the published version of the manuscript.

## Funding

This study was supported by the National Key Research and Development Program of China (No. 2017YFC1104100).

## Conflict of Interest

The authors declare that the research was conducted in the absence of any commercial or financial relationships that could be construed as a potential conflict of interest.

## Publisher's Note

All claims expressed in this article are solely those of the authors and do not necessarily represent those of their affiliated organizations, or those of the publisher, the editors and the reviewers. Any product that may be evaluated in this article, or claim that may be made by its manufacturer, is not guaranteed or endorsed by the publisher.
